# Prospective assessment on the cephalometric profile changes induced by labial hyaluronic acid infiltrations: A before-and-after study

**DOI:** 10.4317/jced.63546

**Published:** 2026-01-28

**Authors:** Pilar Carrero-Brioso, María Baus-Domínguez, Gonzalo Ruiz-de-Leon, Carmen Keim-del-Pino, María Angeles Serrera-Figallo, Celia Vazquez-Pachon, Daniel Torres-Lagares, José Luis Gutierrez-Perez

**Affiliations:** 1 DDS, PhD Student, Department of Dentistry, Dental School, University of Seville; 2 DDS, PhD, Department of Dentistry, Dental School, University of Seville; 3 DDS, DMD, PhD, Department of Dentistry, Dental School, University of Seville; 4 DDS, Master in Oral Surgery, Department of Dentistry, Dental School, University of Seville; 5 DDS, PhD, Full Professor of Oral Surgery and Oral Medicine, Department of Dentistry, Dental School, University of Seville; 6 DDS, PhD, Full Professor of Oral Surgery, Department of Dentistry, Dental School, University of Seville; 7 DMD, PhD, Full Professor of Oral Surgery, Department of Dentistry, Dental School, University of Seville

## Abstract

**Background:**

Facial disharmonies are caused mainly by aging and malocclusions. Hyaluronic acid has been positioned as the dermal filler of choice for volume restoration. This study aimed to assess the effects of hyaluronic acid lip injections on facial profile and their durability.

**Material and Methods:**

A before-and-after study was carried out on 32 patients receiving hyaluronic acid lip infiltrations. A follow-up was scheduled at one-month intervals for three months, and at six months, in which four angular and two linear measurements were evaluated to determine changes in facial profile. These measurements consisted of the angle formed by lines Ls-N and L-N-Po; the angle between lines Li-N and L-N-Po; the angle subtended by lines Ls-Sn and Sn-Col; and the angle constructed from lines Li-Sn and Sn-Col. Likewise, the distance between point Ls and line SL, and the distance between point Li and line SL. Additionally, they were evaluated stratigraphically according to age ( 30 or &gt;30 years) and volume ( 0.7 or &gt; 0.7 mL).

**Results:**

Statistically significant results were obtained. A decrease of 5.76±9.81°, p &lt;0.01, was found for Angle(Ls-Sn/Sn-Col). Additionally, the Distance (Ls-SL) showed a decline of 1.27 ± 2.11 mm, p &lt; 0.01. Concerning the results stratified by infiltrated volume, greater angular changes were observed for volume &gt;0.7ml, as well as for age, an increase of 3.06±6.81°,p&lt;0.05 was gained for Angle(Li-N/N-Po), as a decrease of 2.91±1.80 mm, p&lt;0.05 was observed for Distance(Ls-SL) in patients &gt;30.

**Conclusions:**

The data revealed variability in angular and linear measurements among visits, indicating that final, sustained changes translated into aesthetic improvements. However, these results may vary depending on the volume injected and the patient's age.

## Introduction

Facial attractiveness exerts a significant influence within the social frame. Appearance can influence the response society has towards one and is likely to be perceived as more competent and likable, thereby having an impact on self-esteem and social adjustment. (1) As a multifaceted construct, it is influenced by numerous components. While neoclassical beauty standards are often regarded as the ideal reference, there is variability in what the ideal could be understood to mean, based on a patient's personal desires, age, ethnicity, gender, and individual preferences, considering that modern trends significantly influence perception. Despite the subjectiveness of youthfulness, symmetry, averageness, and sexual dysmorphism, these attributes are universally recognized as key to facial beauty, particularly well-proportioned features that provide harmony and, consequently, overall facial enhancement (1 , 2). To achieve aesthetic outcomes aligned with patient aspirations, the dynamic field of aesthetic medicine and surgery continues evolving. A comprehensive aesthetic evaluation is essential to identify, quantify, and address facial disharmonies or desired modifications (3 - 5). Numerous factors contribute to alterations in the facial profile, with aging and malocclusion being primary determinants. The aging process is characterized by volume depletion and changes in facial contours. Inappropriate restoration of lost volume can paradoxically accentuate an aged appearance; therefore, a comprehensive rejuvenation necessitates a multi-tiered approach that addresses the dermis, subcutaneous fat, musculature, cartilage, and bone as a global approach. For instance, bone resorption and fat loss result in volume loss, while muscle atrophy and hypertrophy contribute to wrinkles and sagging (4 , 6). Concerning our area of interest, the lower third of the face, it is a primary site of age-related changes, especially in the mandible and chin. While facial aging is a holistic process, volumetric loss in the midface, for instance, contributes explicitly to the accentuation of nasolabial folds, jowl formation, and superior lip descent, accompanied by maxillary bone resorption and retrusion. Additionally, preauricular volume depletion exacerbates jowl development and blunting of the mandibular border (5). The lips also undergo age-related changes, including thinning, changes in shape, and loss of volume. Understanding these anatomical changes is crucial for effective facial rejuvenation procedures (6). On the other hand, malocclusions can also provide a lack of harmony of proportions. Notably, in patients with Class III malocclusion, characterized by a concave profile due to skeletal discrepancies presenting a maxillary retrusion and/or mandibular protrusion, often are accompanied by a flattening of the suborbital region extending from the inferior orbital rim to the labial commissure in the bone profile which in soft tissues, manifests as a prominent lower lip and a depressed upper lip. Despite early orthopedic, orthodontic, and even surgical correction, the soft tissue profile may not fully align with the favorable skeletal changes that have occurred. In many cases, additional interventions, such as infiltrations to address labial incompetence in severe cases and enhance convexity, are necessary to reach more optimal outcomes. According to research, the soft tissue response to complex tissue modifications ranges from 50% to 80%. Many figures in orthodontics, such as Holdaway, Angle, Burnstone, Tweet, and Jacobs, among others, have not only contributed with therapeutic proposals, but have also emphasized orofacial harmonization when evaluating and treating their patients (4 , 6). Aiming to restore soft tissue volumes, multiple cosmetic body-shaping procedures can be employed. These can be categorized into two groups: those that remove tissue and those that add volume. For the latter, there are many alternative treatment options, including fat transfer, non-resorbable silicone implants, and injectable products such as silicone, polyalkylimide, and polyacrylamide gels. The most commonly used injectable dermal fillers are the resorbable ones, with hyaluronic acid (HA) being the most employed. The last ones are optimally suitable for facial soft tissue augmentation and can be used to rectify defects ranging from superficial to deeper. Based on the duration of effect, they can be categorized as temporary, semipermanent, and permanent. Additionally, regarding the mechanism of action, as replacement fillers or stimulatory fillers (4 , 7). Filler selection should align with the patient's needs, encompassing subtle contouring to structural reshaping (7). A contemporary trend in orofacial aesthetics involves the use of minimally invasive techniques to enhance orofacial harmony. Hyaluronic acid (HA) is a naturally occurring polysaccharide of glycosaminoglycan kind found in the extracellular matrix of various tissues, including the skin. It was first isolated in the 1970s and approved by the FDA in 1981. It is a resorbable molecule with a mean lifetime range of 6 to 24 months (4). It has emerged as a prominent material in this domain due to its unique properties, such as its ability for water binding, high viscoelasticity and biocompatibility, which positions it as an optimal dermal filler as hydrating agent and collagen regenerator allowing to restore volume, wrinkle attenuation, and contour enhancement so it can be interestingly used as coadjutant treatment for orthodontics applied on the lips (1). It provides predictable, long-lasting results after administration (8 , 9). When it comes to the technique, several approaches have been developed in recent years, parallel to the proliferation of HA (hyaluronic acid) products exhibiting distinct biochemical and rheological properties, each with particular procedural and therapeutic indications (3). Lip augmentation is one of the most commonly sought aesthetic procedures. While the ideal lip remains elusive, it is generally considered to encompass well-defined vermilion borders and a balanced ratio of upper to lower lip fullness and projection, avoiding overcorrection (2). The upper lip extends from the subnasale to the lower free vermilion border of the upper lip superior-inferiorly and nasolabial folds laterally. In comparison, the lower lip extends from the upper free vermillion edge to the lower border of the mandible superoinferiorly and to the commissures of the mouth laterally (6). When evaluating lip aesthetics, from an anatomical perspective, the upper lip is shorter than the lower lip. The upper lip should constitute one-third of the total vertical lip distance, while the lower lip comprises the remaining two-thirds. Moreover, considering the profile view, generally, the upper lip should project slightly more than the lower lip. Ideally, when establishing as reference the line drawn from the Subnasale point (Sn) to the pogonion point (Pg), the upper lip should project 3.5 mm anterior to this line, while the lower lip should project 2.2 mm anterior to it, so the upper lip should project 1-2 mm anteriorly relative to the lower lip (4 , 10 , 11). Additionally, certain elements, such as a V-shaped depression known as Cupid's bow at the center of the upper lip, formed by the peaks of the vermilion border and the central philtrum depression, are crucial to preserve, as they are integral to the natural contours during the infiltration process to avoid an unnatural appearance (11). It is essential to consider that the underlying dentoalveolar structures significantly influence lip projection. The dentition and alveolar ridges provide the foundation for the soft tissues of the lips. When considering lip augmentation, it is essential to maintain a natural tooth show, which is typically 3-4 mm in women (6 , 10). It is mainly necessary to have a balanced relationship among the lips, nose, and chin. Likewise, a profound understanding of the anatomical site, particularly its vascularization, is essential for the successful execution of the technique and the prevention of complications. The superior and inferior labial arteries traverse the depth of the lip, positioned between the orbicularis oris muscle and the mucosa. The arterial system of this region is formed by branches originating from the facial artery, known as the superior and inferior coronary arteries. To mitigate the risk of vascular injury, injections should be restricted to a depth of 3 mm in the mid-dermis. The product is administered at four injection points, two within the upper lip and two within the lower lip. A multilinear, retrograde, fanning technique is employed to target both the lip edge or vermillion border and the mucosal/submucosal junction from a single injection site, as illustrated by Ribé et al. (11). According to the literature, some complications derived from the technique can be defined; early complications, which occur during the first two weeks that reads as ecchymosis, edema, erythema, infection, allergic reaction, edema, necrosis or embolism and the delayed ones from the second week post treatment to one year on, which include angioedema, hyperpigmentation, infection and granulomas. To prevent complications, the following principles are recommended to be followed for safety during the procedure: use of 37 to 30 cc needles, retrograde injections, small syringes (&lt; 1mm3), low-pressure injections, caution in scarred regions or with previous traumatization, awareness of regional anatomy, and hyaluronidase availability (9). The purpose of the present study was to assess the impact of hyaluronic acid lip infiltration on facial profile changes, considering angular and linear measures as primary outcomes.

## Material and Methods

**Figure 1:**
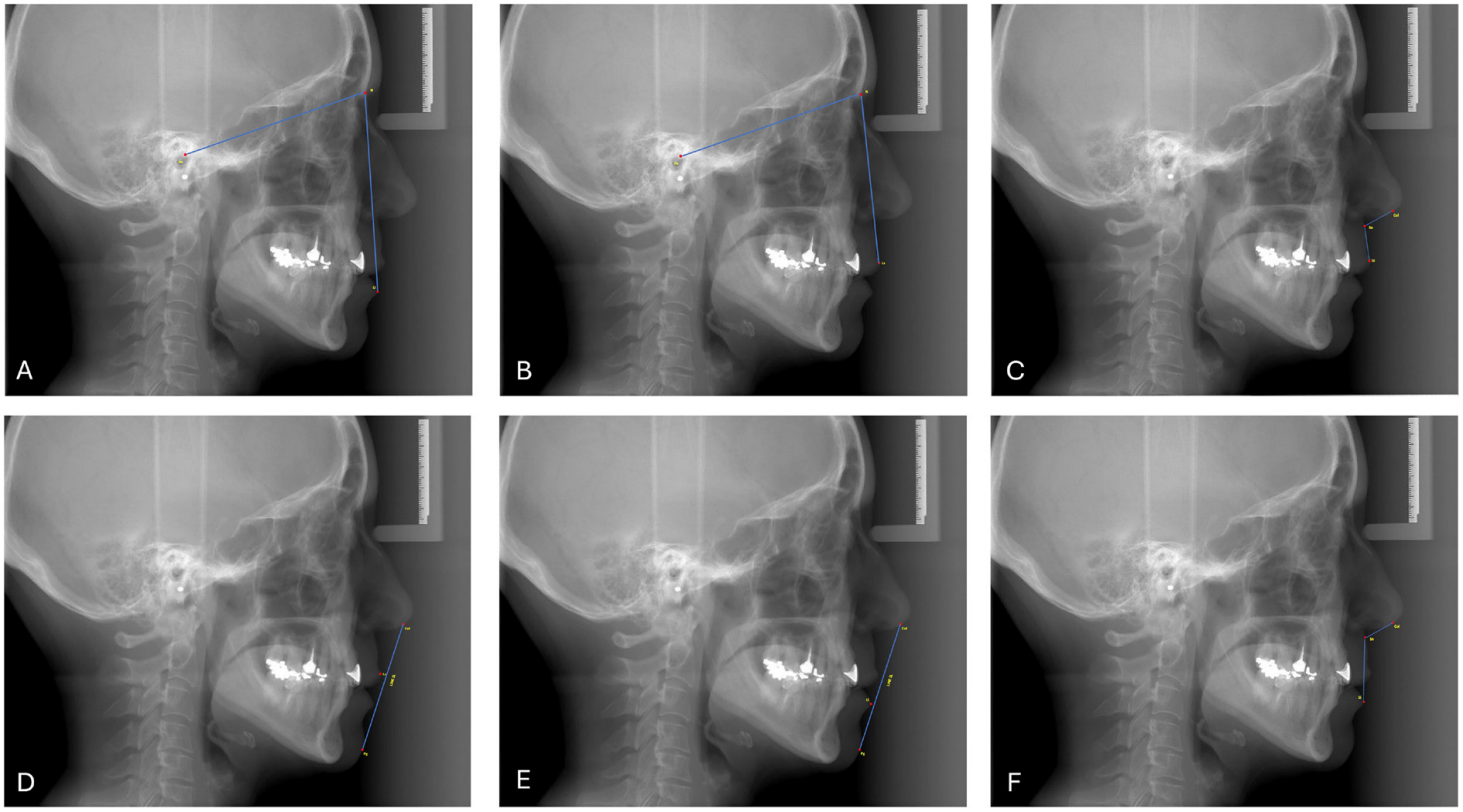
Medidas tomadas en el estudio. A) Angle (Ls-N/N-Po). B) Angle (Li-N/N-Po). C) Angle (Ls-Sn/ Sn-Col). D) Angle (Li-Sn/Sn-Col). E) Distance LS-SL, F) Distance LI-SL.

- Study design This research is a prospective descriptive study applied to a single cohort approved by the Ethics Committee with the code CEIm HM Hospitales 23.11.2258-GHM. It adheres to the ethical principles outlined in the World Medical Association's Declaration of Helsinki: Ethical Principles for Medical Research Involving Human Subjects (12) and follows the STROBE guidelines for cohort studies. All participants were provided with detailed information about the study and gave informed consent for their involvement, including the infiltration of hyaluronic acid and the periodic control radiographs taken for measurements during the follow-up period. - Participants The study was conducted on a sample of 32 patients recruited from the Faculty of Dentistry at the University of Seville who underwent lip hyaluronic acid infiltrations to enhance facial profile harmony. Individualized treatment approaches were necessary due to the unique facial profiles of each patient, requiring specific hyaluronic acid volumes and injection sites, particularly in the lips, whose diagnoses were based on cephalometric studies conducted by expert orthodontists. The criteria for inclusion in the study were as follows: aged 18 to 65 years; feminine sex; heterogeneous cephalometric profile consisting in patients with mild to moderate Angle class I, II, and III malocclusions; history of pre-treatment comprehensive cephalometric profile assessment by a qualified professional, including cephalometric radiographs and clinical photographs; overall good health with no significant medical contraindications to hyaluronic acid treatment. Exclusion criteria were: Age restrictions: people under 18 or over 65 years; severe deformities requiring orthognathic surgery; medical contraindications (allergies, autoimmune diseases, coagulation disorders, active infection at the site, pathological scarring, interference medications); pregnancy and &lt;6 months of hyaluronic acid facial treatments. - Therapy procedure The procedure was carried out using a single syringe of reticulated hyaluronic acid, specifically designed for lip augmentation, containing 1 ml of 23 mg/ml concentration, and a 27 G needle. The exact amount infiltrated was recorded for each patient. The technique could vary individually based on patient needs. Initially, the appropriate needle gauge was selected to determine the point of entry. For lip contouring, the needle was inserted obliquely, nearly parallel to the skin's surface, and the material was deposited retrogradely along the cutaneous-mucosal junction or virtual channel. The procedure commenced approximately 2-3 millimeters from the labial commissure. The lip was gently pinched with the contralateral hand to palpate the injected volume and define the placement along the lip's vermilion border. In case the cupid bow needs to be enhanced, it is infiltrated with material in a V-shape, with two slopes, from a central point. Subsequently, if more contouring was required, the philtrum columns were enhanced by injecting material parallel to the columella, starting from the lip border. At last, in those patients who needed more lip volume, the lip's vermilion and body were filled by depositing material. To elevate the lateral aspects of the lip, the needle was inserted at a steeper angle of 4-5 millimeters, employing a retrograde technique. - Radiographical measurements Before and following the infiltration treatment, all patients underwent angular and linear measurements at each appointment. These measurements were obtained from digital lateral teleradiographs performed using Carestream CS8100 3D equipment. Follow-up teleradiographs were acquired at one, two, three, and six months post-treatment. The primary outcome variables, measured at each of the five appointments, included four angular and two linear measurements, as detailed below and depicted in Figure 1.

1

- Angular measurements Angle (Ls-N/N-Po). It is determined by the angle between the lines respectively conformed from Labrale superius point (LS), defined as the most anterior point of the upper lip, to the Nasion point (N), given as the intersection of the nasofrontal suture and the sagittal midline, being the most anterior point of the frontonasal suture. The line fitted between (N) and the Porion point (Po), which is the most superior point of the external acoustic meatus (Fig. 1A). Angle (Li-N/N-Po). The angle determined by lines respectively conformed from the most anterior point of the lower lip, Labrale inferius (Li), to point (N), and the line from point (N) to point (Po) (Fig. 1B). Angle (Ls-Sn/ Sn-Col). The angle constructed from the line between point (Ls) and the Subnasale point (Sn), defined as the soft tissue point located at the intersection of the upper lip and the nose, and the line conformed from the Sn point, previously described and the Columella point (Col), which is located on the lower surface of the nose, determining the anterior limit of the nasolabial angle (Fig. 1C). Angle (Li-Sn/Sn-Col). The angle between lines formed respectively from point (Li) to point (Sn), and the line between (Sn) point to (Col) point (Fig. 1D). - Lineal measurements Distance LS-SL, defined as the distance between line SL, being conformed from point (Col), to Pogonion soft point (Pg'), referring to the most anterior point of the chin protrusion located on the smooth profile, to point (LS) (Fig. 1E). Distance LI-SL, the distance between SL line conforming from point (Col) to point (Pg') and point (Li) (Fig. 1F). A result was generated for each measurement, comparing the evolution of the profile among visits with the pretreatment measures, subtracting the differences of previous visits to obtain a better understanding of the evolution before and after treatment. - Follow up A follow-up period was defined, consisting of four visits after the initial treatment appointment. The first three visits were scheduled monthly, and the final visit took place six months after treatment. At every visit, the patients were examined, taking the angular and linear measures mentioned above to assess changes over time. - Statistical Analysis A comprehensive descriptive analysis was performed for all angular and linear variables, which were measured quantitatively and expressed as Mean ± Standard deviation (mean ± SD), excluding those who did not complete follow-up. The normality of numerical variables was assessed using the Kolmogorov-Smirnov test. All numerical variables were normally distributed except for Age, the one-month after treatment mean value for the Angle (Li-Sn/Sn-Col), the initial mean value for the Angle (Li-Sn/Sn-Col), the difference mean value two months after treatment for the Angle (Li-Sn/Sn-Col), and the difference mean value six months after treatment for the Angle (Li-Sn/Sn-Col). For comparisons between numerical variables, ANOVA was used for variables with a normal distribution, while the Mann-Whitney U test was used for variables with a non-normal distribution. Statistical significance was set at p &lt; 0.05. Subgroup stratified analyses were carried out, categorizing patients into two groups based on age (30 years and &gt;30 years) and on the volume of hyaluronic acid injected (0.7 mL and &gt;0.7 mL).

## Results

The original sample size was calculated to be 24 patients who fully comply with the follow-up protocol. However, to mitigate the risk of attrition bias, an additional eight patients were recruited, bringing the total sample size to 32. Accepting an alpha risk of 0.05 and a statistical power greater than 0.8 in a unilateral contrast, 13 subjects are required to detect a difference equal to or greater than 1 unit as statistically significant. A standard deviation of 1 is estimated for the first measurement and 1 for the second measurement. It has been calculated, assuming a correlation coefficient of 0. A loss-to-follow-up rate of 0% is estimated. This ensured that the final sample size was sufficient to draw reliable conclusions from the study. All the participants were women with a mean age of 32,59±13.35 with a range from 25 to 40 years. Regarding the infiltrated volume, the mean volume was 0.94 ± 1.15 mL, ranging from 0.5 to 2 mL. - Descriptive analyses Before-and-after outcomes Angular measures Regarding the Angle (Ls-N/N-Po), minimal fluctuations were observed in the first month post-treatment. A modest increase of approximately one degree occurred in the second month compared to the baseline measurement. Subsequently, a decrease of this value was noted in the third month, and ultimately, a maximum average increase of 1.37 ± 3.95° was reached at the six-month follow-up, yielding a final value of 16.83 ± 4.65°. Regarding the Angle (Li-N/N-Po), it exhibited minimal variability across the different appointments. The only notable exception was an average increase of 1.05 ± 4.61° in the second month. Ultimately, a slight average growth of 0.36 ± 5.22° was observed at the final visit six months after treatment. Concerning the Angle (Ls-Sn/Sn-Col), the mean measure pre-treatment was 106.98±9.79°, and it underwent a moderate decrease one month later, with a difference of -8.86±10.66°, which was statistically significant with a p-value &lt; 0.001. While a slight increase in the value occurred in the second month, it was followed by a modest decrease in the third month. Finally, the six-month measurement was an average of 101.42 ± 10.77°, representing a statistically significant average decrease of 5.76 ± 9.81° compared to the initial average value (p &lt; 0.01). In contrast, the Angle (Li-Sn/Sn-Col) exhibited a rebound at the six-month appointment, reaching an average value of 122.12 ± 37.25 degrees. This represents a 5.33 ± 25.21° average increase in comparison to the initial average measurement. The mean differences of these variables are all shown in Table 1.

Table 1

- Lineal measures The distance Ls-SL decreased from the initial measurement to the six-month measurement. A statistically significant average decrease of 1.61 ± 1.82 mm was observed at the two-month follow-up (p &lt; 0.001). While a slight increase occurred at the three-month appointment, a subsequent average decrease was noted at the six-month appointment, resulting in a statistically significant average reduction of 1.27 ± 2.11 mm from the initial measurement (p &lt; 0.01). Meanwhile, the distance Li-SL exhibited a slight decrease from the initial to the second month post-treatment. However, a modest increase was observed at the six-month appointment, resulting in a minimal 0.05 ± 1.32 mm average increase compared to the baseline measurement, as shown in Table 1. - Outcomes from stratified analyses by subgroups according to age The mean age of the 32 patients was 32.59 ± 13.35 years. Therefore, the cut-off point was established at 30, corresponding to the median age of the sample. This determined the two study groups to be 30 years and &gt;30 years. To account for potential age-related confounding factors. The first group presented a total of 22 patients, representing 68.8% of the sample, while the second group presented a total of 10 patients, representing 31.3% of the sample. Angular variables For the variable Angle (Ls-N/N-Po) in the group 30, a progressive pattern was not observed, with an average rebound of 2.08±2.96 ° in the second month after the treatment appointment and a slight average decrease at the six-month post-treatment appointment, resulting in a final average increase of 1.58±4.32° compared to the first visit. In contrast, as it can be observed in table 2, the group of patients &gt;30, despite showing a similar measurement at the first visit, its value decreased until the third month post-treatment, experiencing a slight average increase at the six-month appointment of 0.95±3.23°, being a proportionally lower value acquired than in the group of patients 30. The Angle (Li-N/N-Po) variable shows a progressive decrease in variation among patients 30 throughout the appointments, with a final mean value of 30.81±6.83 ° compared to the initial visit's mean value of 32.43±7.16°. In comparison, in the group of patients &gt;30, despite presenting a lower mean value than the initial one belonging to the group 30, there was an upward trend of a mean difference of 3.06±6,81°(p&lt;0,05) higher than the mean value assessed at the initial visit and higher than the final mean value obtained from the group of patients 30 years as it is represented in Table 2.

Table 2

Regarding the Angle (Ls-Sn/ Sn-Col) variable, the group of patients 30 had a mean measurement of 108.13±8.11 ° after the first infiltration, experiencing a decrease in the first month post-treatment and a slight increase in the second month post-treatment. It ended with a mean value of -5.14 ± 8.22 °C lower than the initial mean value. The group of patients &gt;30 presented at first a lower mean value than the group 30 being 104.46±12.90°, however, following the same trend, as it is exposed in table 2, the value presented changes between appointments finally obtaining a mean value of -6.99±12.85° lower than the initial value of the same group, being also lower than the final mean value acquired by the group 30. At last, the Angle (Li-Sn/Sn-Col) presented little variances in the group 30, with a slight spike at the third month of 6.62±30.99°. Finally, its value increased by 0.56±8.58° compared to the first visit. On the other hand, in the group of patients over 30, this variable behaved more randomly, exhibiting an increase at the second visit, a decrease at the third month, and a progressive rise at the last visit, where it achieved a mean difference of 14.87±41.72°, obtaining its highest value. Lineal variables The distance Ls-SL, as presented in Table 2, acquired a lower mean value at the initial visit in the group 30, being 3.39 ± 2.37 mm, compared to the group &gt; 30, which had a mean value of 4.80 ± 2.42 mm. Both groups showed a downward trend, with a slight decrease at the last appointment compared to the initial visit in the group under 30, as reflected in the mean value with a difference of -0.81±2.07 mm. The group over 30 showed a similar trend, with a mean value difference of -2.20±1.96 mm compared to the initial visit. Distance Li-SL, whose values are also outlined in Table 2, did not present a progressive trend in any direction throughout the visits, exhibiting in ten groups 30 a decrease of the mean value at the second month of just -0.01±0.95 mm and a slight increase at the final one, with the average difference only of 0.11±1.18 mm. On the other hand, although the group &gt;30 presented a value in the initial visit higher than the initial value of the 30, it grants a fluctuation with a pattern similar to the group 30, concurring to a decrease reached at the second month of -1.05±1.72 mm finishing with an average difference to the initial visit of -0.07±1.62 mm. - Outcomes from stratified analyses by subgroups according to volume of hyaluronic acid infiltrated. To ensure accurate analysis, two study groups were established based on the initial volume of product infiltrated. A cut-off point of 0.7 ml was selected, aligning with the mean volume administered (0.7 ml). This division resulted in two groups: a low-dose group ( 0.7 ml) and a high-dose group (&gt; 0.7 ml), each comprising 16 patients (50% of the total sample). This stratification aimed to mitigate the potential influence of volume-related confounding factors on the study outcomes. Angular variables The Angle (Ls-N/N-Po), as depicted in Table 3, reached a proportionally similar final difference balance in both groups.

Table 3

In the group 0.7 ml, the initial average value was 14.54±4.38°, and it finished with an average value of 15.55±3.69°. Similarly, in the group &gt;0.7 ml, the final mean value obtained was 17.33±4.18 °, with an initial mean value of 16.24±5.00 °. This implies a slightly higher value in the group &gt;0.7 ml compared to the 0.7 ml group. Concerning the Angle (Li-N/N-Po), the group 0.7 ml presented a slight increase at the last visit in comparison to the initial visit, with a mean difference of 0.52±5.08°, while the group &gt;0.7 ml experienced an average decrease of-0.32±5.67° compared to the initial visit, as it is expressed in Table 3. Concerning the Angle (Ls-Sn/Sn-Col), a similar tendency was observed in both groups, with a greater value in the group &gt;0.7 ml and a lower mean final value, differing by an average of -9.14±8.89° compared to the initial average measure. The mean difference in the group, 0.7 ml, between the initial and last visit was just an average of -1.86±10.21°. The Angle (Li-Sn/Sn-Col) behaved similarly, presenting an increase in both groups, with a proportionally greater increase in the &gt;0.7 ml group, showing a difference of 9.77±36.12° between the last and initial appointments. In the 0.7 ml group, the tendency during the appointments was progressively slighly increasing, except in the second month when it presented an average difference increased of 11.59±35.59° and then reduced considerably in the third month post-treatment to a more stable final mean value in the six-month appointment with an average difference of only 1.29±7.82° to the initial visit as it is indicated in Table 3. Lineal variables The distance Ls-SL exhibited similar variability in both groups, as shown in Table 3, with the average difference slightly higher in the group with a value greater than 0.7 ml, at a mean difference of -1.47±2.17 mm. The initial average value in the group 0.7 ml was 4.37±2.51 mm in comparison to the average value of 3,60±2,39 mm from the group &gt;0.7 ml. Although the average difference reached is a higher value in the group &gt;0.7 ml, the mean value at the end of the study was still higher in the group 0.7 ml, with a mean value of 3.36±1.65 mm, compared to the mean value of the group &gt;0.7 ml, which was 2.00±1.89 mm. The distance Li-SL displayed a very slight variation along the visits in both groups, presenting a progressive recession, experiencing a very slight increase in the last visit in the group &gt;0.7 ml with a mean difference of 0.07±1.09 mm, and an imperceptible decrease in the group 0.7 ml with a mean difference of -0.17±1.44 mm.

## Discussion

Hyaluronic acid fillers have become a popular aesthetic treatment for lip augmentation, as reflected in the literature. They offer immediate results and provide a safe, effective, and reversible treatment option. It's gaining increasing significance worldwide, with over 2 million people opting for hyaluronic acid fillers as their preferred choice for lip harmonization (13 , 14). Although its primary aim is to enhance lip volume and shape, its impact on the overall facial profile is well-known. Many factors can influence the fluctuation of how hyaluronic acid affects the facial profile over time (14). Given the resorbable molecule's inherent variability in volume maintenance, as evidenced by the experimental data, accurately predicting its future behavior remains a complex challenge. The current study focuses on investigating how hyaluronic acid behaves over time in terms of lip volume preservation, analyzing its relationship to other facial structures, especially the chin and nose, as previously exposed (15 , 16). Multiple systematic reviews and clinical trials have demonstrated that strategic hyaluronic acid injection techniques can subtly alter facial proportions and achieve facial balance (6 , 14 - 17). For instance, increasing lip volume can accentuate the philtrum, soften the nasolabial folds, and improve the projection of the lower face. However, excessive or poorly placed injections can lead to undesirable outcomes, such as a distorted lip shape, an overfilled appearance, or a "duck-lip" effect (1 - 3). We decided to employ quantifiable metrics that can be visually discerned as alterations in the overall facial profile to objectively determine the extent of these modifications, which can range from minimal to substantial. We considered teleradiographs as standardized and calibrated images for all patients, offering a reliable and objective tool for measurement. To date, only two systematic reviews have explored the efficacy and safety of hyaluronic acid fillers for lip augmentation: those by Cohen et al. (16) and Stojanovi and Majdi (17). Both reviews demonstrated the effectiveness of HA fillers in increasing lip fullness over time. However, a limitation of these reviews was indeed the significant variability in the scales used to measure and to report data across primary-level studies, hindering direct comparisons. In 2021, a meta-analysis conducted by Czumbel LM et al. (15) demonstrated that lip augmentation with HA could provide significant and sustained improvements in lip fullness. Employing the same follow-up as we did in our study, they reported at the two-month follow-up a 91% improvement in lip fullness of at least one grade, which was slightly reduced to 71% maintained volume of at least one grade improvement in lip fullness at three months post-injection. While this positive effect persisted at six months, with 74% of participants still showing improvement, even twelve months after treatment, 46% of participants continued to experience benefits from the initial treatment. Our findings corroborate these facts. In our study we measured and analyzed the chages of different angles regarded as important parameters specifically, the Angle (Ls-N/N-Po), which assesses the upper lip projection relative to the cranial base, which increased from a mean of 15.45 ± 4.59° pre-treatment to a mean of 16.83 ± 4.65° at the six-months post-treatment appointment, reaching its maximum value. It can be considered a moderate protusion of the upper lip. Conversely, the angle (Li-N/N-Po), which measures lower lip projection relative to the cranial base, demonstrated a less pronounced increase, with a mean difference of 0.36 ± 5.22° at the six-month appointment. This is consistent with the general aesthetic principle of the upper lip projecting slightly more than the lower lip. Furthermore, the reading of these results is corroborated with more strength when the angles that determine the relationship between the lips and the nasal base are evaluated. As it has been employed in other studies (18 , 19), on the one hand, the relation between the upper lip and the nasal base would be examined by the angle (Ls-Sn/Sn-Col), which is the most frequently used soft tissue parameter in orthodontic diagnosis, representing the inclination of the upper lip. An obtuse angle, meaning a larger value of the angle, would indicate a depression of the upper lip, regarded as the typical appearance of an aged face. A more pleasant and youthful appearance can be appreciated when the distance from the nose to the vermilion border of the upper lip shortens and the visualized quantity of the lip vermilion increases (20), so a lower value might indicate a more prominent upper lip. One month after treatment, its maximum prominence was reached, with a mean decrease of 8.86±10.66 degrees, which was statistically significant (p &lt; 0.001). Over time, the hyaluronic acid filler underwent a process of resorption, so the mean value of this angle slightly increased, as expected. Thus, at the six-month appointment, a remarkable mean difference of 5.76±9.81° was reached, which was newly statistically significant (p &lt; 0.01). Hence, despite its resorption, a notable prominence of the upper lip was observed. As previously stated, the chin, in its relationship with the lips, is a key element in assessing facial profile (11 , 21). Thus, on these terms, the line (SL) drawn from the Columella point (Col) to the soft pogonion (Pg') can serve as a reference line to determine the ideal projection of both lips by the chin. A shorter distance from (Ls) or (Li) to line SL would imply a more prominent lip, while a larger distance would shelter a more depressed lip. The distance from (Ls) to line SL, decreased 1.27±2.11 mm six months after treatment, being a statistically significant result (p&lt;0.01), which would be translated into a prominence of the upper lip whilst the distance from the lower lip to this reference line increased at the six-month appointment very slightly, with a mean difference value of 0.05±1.32 mm despite decreasing along all the previous follow-up appointments. This data should be taken carefully, as it may be subject to an information bias that could be attributed to the multiple individuals involved in data collection and management. As mentioned earlier, numerous confounding factors can influence the rate of hyaluronic acid resorption. We focused in depth on two key confounding factors-age and volume of product infiltrated-to assess their potential impact on outcomes. However, we are aware that other patient-specific factors, such as smoking, hydration, and sun exposure, may also influence outcomes. Aging is a complex process influenced by both genetic and environmental factors (6). Perioral and lip aging is a multifactorial process, with factors interrelated among them, which implies a problematic understanding of their real implication on the longevity of hyaluronic acid. External and internal soft tissue changes, including atrophy of the philtrum, the columella, and the orbicularis muscle, contribute to a more depressed upper lip. Consequently, rejuvenation procedures often necessitate addressing the surrounding structures, such as the premaxilla, nose, chin, and perioral region, structures that remained untouchable in the patients involved in this study, so it can negatively influence the final esthetic profile of these patients, as the infiltration treatment was strictly applied just on the lips. The angle (Ls-N/N-Po) increased moderately in both groups, with an average difference of almost one degree (0.95±3.23°) in the group of patients over 30 at the six-month post-treatment appointment. In comparison, in the group 30, a half degree more average difference was achieved, (1.58±4.32°), with the particularity that both groups started with a similar initial average value, which suggests a greater maintenance of upper lip prominence in both groups. It is remarkable to note that the angle (Li-N/N-Po) decreased by almost one degree in the group 30, with an average difference of -0.99±3.72° at the six-month post-treatment appointment, indicating a more depressed lower lip than initially. In contrast, in the group of patients over 30, it rises considerably, with a mean difference value of 3.06±6.81, which is statistically significant (p&lt;0.05), suggesting a more prominent lower lip achieved six months post-treatment. Regarding the angle (Ls-Sn/Sn-Col) it would be expected to increase with age due to the decreased support and projection of the soft tissue envelope, leading to the loss of vermilion, however in both groups it experimented a decrease of their respective average values being greater, surprisingly, in the group of patients &gt;30, which started from a lower initial mean value which would suggest a more prominent upper lip initially and at the six months than the mean value, both initial and final, belonging the group 30. This group obtained a mean value of 103.40±10.44°, compared to the mean value of the &gt;30 group, which was 97.47±10.85°. On the contrary, the angle (Li-Sn/Sn-Col), which would be expected to decrease to imply a more prominent lower lip showed a little increased mean value in the group of patients 30 and a massive rise in the group &gt;30 that we seriously consider to be an error of measurement counting with a mean difference of 14.87±41.72°. The distance Ls-SL decreased in both groups, being greater in the group of patients over 30, resulting in a more prominent upper lip. On the other hand, distance Li-SL increased slightly in the group 30, which would result in a retrusion of the lower lip six months after treatment. In the group of more than 30, this value decreased and remained uniform throughout the appointments. Hence, after evaluating this data, as the data obtained did not show statistical significance, conclusions are not able to be throw so easily; even though the age is hypothesized to be an essential factor that can modify the behave of hyaluronic acid as it is believed that age-related changes in cellular metabolism can slow down the body's natural healing process, impacting the integration of the filler and its longevity, we observed more favorable aesthetic results in the &gt;30 group. Concerning the volume of HA injected, it was expected that the group receiving &gt;0.7 ml would exhibit more favorable outcomes compared to the group receiving 0.7 ml. This is because, after a similar period, a larger residual amount of HA would remain in the lips, as the rate of resorption would be proportionally equal. Angle (Ls-N/N-Po) showed a more increased value in the group 0.7 ml than in the group &gt;0.7 ml, although as the initial mean measure was wider in the group &gt;0.7 ml, it obtained a wider angle average value at the six-month appointment of 17,33±4,18°, meaning a more prominent upper lip as expected. The angle (Li-N/N-Po) did not show considerable differences that could be observed clinically; however, unexpectedly, this angle decreased in the group with a volume greater than 0.7 mL, with a final average value of 32.02 ± 6.20 °. The Angle (Ls-Sn/ Sn-Col) confirmed this assumption as the decrease of this angle was much larger in the group &gt;0.7 ml than in the group 0.7 ml, obtaining a final average value of 100,13±11,17°. The distance Ls-SL decreased in both groups, indicating that an improvement in this value resulted in a more prominent upper lip at the end of the follow-up period. Likewise, a larger decrease in distance was observed in the group &gt;0.7 ml, with an average value of 2.00±1.89 mm, showing a more prominent upper lip than in the group 0.7 ml. These data should be taken carefully, considering the small sample size and the information bias likely introduced due to the multiple practitioners who collaborated in the study. Although HA is regarded as an adequate soft tissue filler, it still has limitations related to the ease of injection and its persistence. The results depend on the technique employed, the level of expertise, and the practitioner's experience. Additionally, it is essential to select the correct product for each situation individually, so a thorough knowledge of the chemical and physical characteristics of the formulation is required (21). To standardize the technique, all patients were infiltrated with the same HA product. This may have compromised the statistical power, leading to underpowered results. It should also take into account the lack of consensus on a standardized, validated scale for assessing the efficacy and longevity of HA, which would enable comparison of results across studies (22). Another limitation of this study is its static nature. The lips and the perioral area are highly mobile, and it is suggested that they be more appropriately evaluated with dynamic evaluation, as demonstrated by a recent study that measured the "stretch" of a 3-D perioral surface. Future studies may compare 3-D volumetric changes with qualitative and dynamic changes in lip aesthetics (4 , 23). Future research should elucidate whether other factors, apart from the age and volume of hyaluronic acid, can act as cofounding factors and enhance or undermine the potential of hyaluronic acid to have long-term effects on facial aesthetics, including potential changes in tissue quality and collagen production. Additionally, studies exploring the optimal injection techniques and product selection for various facial types and aging patterns would be valuable. Higher-quality evidence publications are needed in the form of systematic reviews and meta-analyses evaluating this matter. We encourage authors to conduct clinical studies using standardized measurement systems to ensure comparability among them and reduce the risk of bias. Forthcoming studies with more participants with a wide age range and different ethnicity or gender are suggested to validate our findings further, as HA injections have traditionally been a predominantly female aesthetic practice. However, as society evolves, the increasing blurring of gender lines has prompted men to consider these procedures more often increasingly.

## Conclusions

In conclusion, HA lip fillers, when administered judiciously, can be a powerful tool for enhancing facial aesthetics and a complementary adjunct to orthodontic treatments. By understanding the intricate interplay between lip volume, facial proportions, and aging, practitioners can achieve natural and harmonious results that complement the patient's overall appearance.

## Figures and Tables

**Table 1:** Outcomes for linear and angular variables.

Lineal variables	Measures [mean±SD (mm), p value]				
	Raw data	Difference to the initial measure			
	Initial	Month 1	Month 2	Month 3	Month 6
Distance Ls-SL	3.83±2.44	-1.58±2.13, <0.01*	-1.61±1.82, <0.001*	-1.14±2,17, <0.01*	-1.27±2.11, <0.01*
Distance Li-SL	1.75±1.47	-0.06±1.40	-0.31±1.28	-0.21±1.59	0.05±1.32
Angular variables	Measures [mean±SD* (°), p value]				
	Raw data	Difference to the initial measure			
	Initial	Month 1	Month 2	Month 3	Month 6
Angle (Ls-N/N-Po)	15.45±4.59	0.72±3.17	1.32±3.96	0.32±3.84	1.37±3.95
Angle (Li-N/N-Po)	31.76±6.76	0.69±4.36	1.05±4.61	0.13±4.65	0.36±5.22
Angle (Ls-Sn/ Sn-Col)	106.98±9.79	-8.86±10.66, <0.001*	-4.93±7.52, <0.01*	-5.63±8.90, <0.01*	-5.76±9.81, <0.01*
Angle (Li-Sn/Sn-Col)	116.47±24.19	0.14±9.93	4.94±26.13	0.17±8.27	5.33±25.21

1

**Table 2:** Outcomes from stratified analyses by subgroups according to age for linear and angular variables.

Lineal variables	Subgroups analyses by age										
	Age ≤30					Age>30					
	Measures [mean±SD (mm), p value]					Measures [mean±SD (mm), p value]					
	Raw data	Difference to the initial measure				Raw data		Difference to the initial measure			
	Initial	Month 1	Month 2	Month 3	Month 6	Initial	Month 1	Month 2		Month 3	Month 6
Distance Ls-SL	3.39±2.37	-1.45±2.46	-1.08±1.58	-0.87±2.06	-0.81±2.07	4.80±2.42	-1.87±1.22	-2.91±1.80, p<0.05*		-1.66±2.40	-2.20±1.96
Distance Li-SL	1.72±1.32	0.06±0.75	-0.01±0.95	-0.09±1.23	0.11±1.18	1.83±1.83	-0.30±2.29	-1.05±1.72		-0.43±2.19	-0.07±1.62
Angular variables	Subgroups analyses by age										
	Age ≤30					Age>30					
	Measures [mean±SD (°), p value]					Measures [mean±SD (°), p value]					
	Raw data	Difference to the initial measure				Raw data			Difference to the initial measure		
	Initial	Month 1	Month 2	Month 3	Month 6	Initial	Month 1		Month 2	Month 3	Month 6
Angle (Ls-N/N-Po)	15.44±4.26	1.01±2.68	2.08±2,96	0.57±3.26	1.58±4.32	15.47±5.50	0.12±4.15		-0.53±5.59	-0.16±4.93	0.95±3.23
Angle (Li-N/N-Po)	32.43±7.16	0.21±4.52	-0.04±4.51	-0,48±4.44	-0.99±3.72	30.29±5.86	1.71±4.05		3.69±3.97	1.28±5.06	3.06±6,81, p<0.05*
Angle (Ls-Sn/ Sn-Col)	108.13±8.11	-8.94±10.20	-5.20±7.22	-5.63±9.01	-5.14±8.22	104.46±12.90	-8.71±12.21		-4.27±8.76	-5.62±9.17	-6.99±12.85
Angle (Li-Sn/Sn-Col)	114.34±8.90	-0.28±10.22	6.62±30.99	1.15±9.11	0.56±8.58	121.15±42.37	1.02±9.83		0.84±5.35	-1.70±6.39	14.87±41.72

2

**Table 3:** Outcomes from stratified analyses by subgroups according to the volume infiltrated for lineal and angular variables.

Lineal variables	Subgroups analyses by infiltrated volume										
	Infiltrated volume≤0,700 ml.					Infiltrated volume >0,700 ml.					
	Measures [mean±SD (mm), p value]					Measures [mean±SD (mm), p value]					
	Raw data	Difference to the initial measure				Raw data		Difference to the initial measure			
	Initial	Month 1	Month 2	Month 3	Month 6	Initial	Month 1		Month 2	Month 3	Month 6
Distance Ls-SL	4.37±2.51	-2.09±2.32	-1.81±1.82	-1.65±2.00	-1.16±2.25	3.60±2.39	-1.20±2.10		-1.43±1.97	-0.73±2.45	-1.47±2.17
Distance Li-SL	1.58±1.63	-0.33±1.76	-0.25±1.45	-0.41±1.83	-0.17±1.44	2.12±1.28	-0.04±0.90		-0.45±1.17	-0.22±1.41	0.07±1.09
Angular variables	Subgroups analyses by infiltrated volume										
	nfiltrated volume≤0,700 ml.					Infiltrated volume>0,700 ml.					
	Measures [mean±SD (°), p value]					Measures [mean±SD (°), p value]					
	Raw data	Difference to the initial measure				Raw data		Difference to the initial measure			
	Initial	Month 1	Month 2	Month 3	Month 6	Initial	Month 1		Month 2	Month 3	Month 6
Angle (Ls-N/N-Po)	14.54±4.38	0.52±3.56	1.25±5.06	-023±4.03	1.24±3.59	16.24±5.00	0.69±3.07		1.48±2.80	0.42±3.40	0.84±3.61
Angle (Li-N/N-Po)	31.44±8.40	0.17±4.27	1.04±6.01	1.02±5.76	0.52±5.08	32.52±5.32	0.83±4.24		1.07±3.04	-0.56±3.78	-0.32±5.67
Angle (Ls-Sn/ Sn-Col)	104.73±9.93	-4.87±8.56	-3.79±6.96	-3.27±8.85	-1.86±10.21	108.37±9.99	-10.50±11.24		-5.96±8.57	-7.27±8.56	-9.14±8.89
Angle (Li-Sn/Sn-Col)	109.92±10.79	0.46±7.83	11.59±35.59	0.79±7.70	1.29±7.82	114.53±9.21	0.36±9.32		-1.42±8.36	-0.22±9.51	9.77±36.12

3

## Data Availability

Data is contained within the article or supplementary material.
